# Links between autobiographical memory richness and temporal discounting in older adults

**DOI:** 10.1038/s41598-020-63373-1

**Published:** 2020-04-14

**Authors:** Karolina M. Lempert, Kameron A. MacNear, David A. Wolk, Joseph W. Kable

**Affiliations:** 1 Department of Psychology, University of Pennsylvania, Philadelphia, Pennsylvania US; 2 Department of Neurology, University of Pennsylvania, Philadelphia, Pennsylvania US

**Keywords:** Psychology, Human behaviour, Cognitive ageing, Cognitive neuroscience, Decision

## Abstract

When making choices between smaller, sooner rewards and larger, later ones, people tend to discount future outcomes. Individual differences in temporal discounting in older adults have been associated with episodic memory abilities and entorhinal cortical thickness. The cause of this association between better memory and more future-oriented choice remains unclear, however. One possibility is that people with perceptually richer recollections are more patient because they also imagine the future more vividly. Alternatively, perhaps people whose memories focus more on the meaning of events (i.e., are more “gist-based”) show reduced temporal discounting, since imagining the future depends on interactions between semantic and episodic memory. We examined which categories of episodic details – perception-based or gist-based – are associated with temporal discounting in older adults. Older adults whose autobiographical memories were richer in perception-based details showed reduced temporal discounting. Furthermore, in an exploratory neuroanatomical analysis, both discount rates and perception-based details correlated with entorhinal cortical thickness. Retrieving autobiographical memories before choice did not affect temporal discounting, however, suggesting that activating episodic memory circuitry at the time of choice is insufficient to alter discounting in older adults. These findings elucidate the role of episodic memory in decision making, which will inform interventions to nudge intertemporal choices.

## Introduction

Throughout our lives, we face many intertemporal choices that involve trade-offs between smaller, immediate gains and larger, long-term benefits^1^. For example, an individual may have to decide whether or not it is worth paying a fee to withdraw retirement funds early. People generally tend to prefer immediate rewards to delayed ones, and they discount the value of delayed rewards to a greater extent as the delay to receiving them increases. This tendency towards temporal discounting is nearly universal, but the rate at which people discount future rewards varies widely^2,3^. Steep temporal discounting, or overvaluing the present at one’s long-term expense, is associated with risky behaviors such as alcohol use^4^, gambling^5^, smoking^6,7^, and excessive credit card borrowing^8^. Given the substantial negative impact of steep temporal discounting, it is important to understand the neurocognitive mechanisms that underlie intertemporal choices, and to use this knowledge to develop interventions that could reduce impatience.

The literature on the neural correlates of temporal discounting points to the involvement of valuation regions^9–11^, executive control regions^12–15^ and episodic memory regions^16,17^ in this choice process. It remains unclear, however, which of these neurocognitive systems underlies *individual differences* in the tendency to discount future rewards. One neurocognitive system that may support future-oriented intertemporal choices is episodic memory, or context-rich memory for autobiographical events^18^. It has been proposed that the ability to retrieve detailed episodic memories could enable a more vivid imagination of the future, which would then enhance the value of future rewards^19,20^. A population that is particularly well-suited for studying the relationship between episodic memory and temporal discounting are older adults. Older adults have substantial variance in their episodic memory function, given that this system declines as people age^21,22^ at rates that vary across individuals^23–25^. Meanwhile, economic decision making (including temporal discounting^26,27^) remains largely stable in older adults^28,29^, suggesting that any changes in economic decision making with aging are underpinned by changes in cognitive abilities^30^. Consistent with this idea, in a recent study we conducted in older adults, we found that lower temporal discounting rates were associated with better episodic memory recall abilities^31^. Temporal discounting was also associated with the structural integrity of a key neural region in the episodic memory system, the medial temporal lobe (specifically, in the entorhinal cortex)^31^. One limitation of this previous study, however, is that it examined only standard neuropsychological measures of memory (word list recall, logical memory, and visuospatial memory recall), which may not fully capture age-related decline in episodic memory in cognitively normal older adults. We were also unable to establish whether semantic or episodic memory was more strongly associated with temporal discounting, since episodic recall in these tasks was likely aided by conceptual knowledge.

One important aspect of episodic memory that may be related to decision making about the future, but is not captured by standard neuropsychological tests, is episodic memory richness. For example, two people might perform equally well on a word list recall task, but they might differ in how vividly they imagined the words on the list at retrieval. In order to assess episodic memory richness in the current study, we examined participants’ *autobiographical* memories, which have been shown to vary in how much detail and what kinds of details they contain^32^. So far, studies that have investigated the link between autobiographical memory richness and temporal discounting have yielded null results^33,34^. It is possible, however, that these studies failed to detect an association because they used a *composite* measure of episodic details that conflates qualitatively different types of details. Mental representations of events (either autobiographical or imagined) are most likely constructed^20^ by binding together different kinds of details that fall into two overarching categories: central, event-related details that make up the “story line” of a memory (previously referred to as “gist-based” details by Sheldon and colleagues^35^), and perceptually rich details that reflect mental construction of a scene (referred to as “perception-based” details by Sheldon and colleagues)^35–37^. Importantly, both categories of details are classified as *internal* to an event, or episode-specific. They differ in that perception-based details suggest that a specific spatiotemporal context is being brought to mind, whereas gist-based details can be detached from their spatiotemporal context.

Which, if any, of these categories of details is associated with temporal discounting? The answer to this question may shed some light on the mechanism by which better episodic memory leads to more patient choice. One possibility is that perception-based details will be linked with lower discount rates. People who can bring to mind the specific time and place of past events, as well as specific objects and sensations from those events, will likely have more detailed simulations of future events as well^38,39^. These more detailed simulations could make future rewards more valuable by making them seem more concrete, certain, and closer in time^40^. Consistent with this notion, studies show that imagining the future more concretely at the time of intertemporal choice increases the likelihood of choosing larger, later rewards^16,17,41,42^. Another possibility is that more future-oriented choice will be associated with the extent to which memories are more gist-based. Event-related details, or gist-based details (to use the terminology of Sheldon and colleagues^35^), are related to the overall *meaning* of an event, including what transpired and in what sequence^43^. They are still episodic, but they are often relatively more “semanticized” compared to perception-based details, since they take longer to decay^36,44^ and rely more on interactions with semantic memory, including event schemas^43,45^. Therefore, people whose recollections feature more gist-based details might more efficiently transform episodic memories into semantic ones^46,47^. Imagining and deciding about the future also relies heavily on efficient interactions between episodic and semantic memory^48–50^; in episodic future thinking, general and personal knowledge provides the “scaffolding” onto which episodic information can be integrated^48^. In line with this hypothesis, individuals with semantic dementia have difficulty imagining novel future events^51^ and have increased discount rates, even compared to individuals with other forms of dementia^52,53^ or hippocampal amnesia^54,55^. Here we used the Autobiographical Interview scoring protocol^32^ to score older adults’ memory descriptions. This protocol identifies five detail categories: time, place, perceptual, event, and emotion/thought details. As in previous research from Sheldon and colleagues^35^, we classified time, place, and perceptual details as “perception-based,” and event and emotion/thought details as “gist-based.” We examined the relationship between temporal discounting and the extent to which autobiographical memories were richer in perception-based or gist-based details, as well as the number of internal details in memory descriptions overall.

A secondary, exploratory aim of this study was to examine, in a subset of participants who had structural neuroimaging data, how different categories of autobiographical details, as well as temporal discounting, are associated with the structure of different subregions of the medial temporal lobe (MTL). We were especially interested in interrelationships between entorhinal cortex (ERC) thickness, autobiographical details, and temporal discounting, since ERC thickness is correlated with temporal discounting^31,56^. The extent to which ERC thickness is associated with perception-based vs. gist-based details may reveal how ERC contributes to more patient choice. The ERC is important for integrating spatial and contextual information into episodic memory^57,58^, suggesting that it may be associated with perception-based details. At the same time, the ERC is also a “relay station” connecting hippocampus with the prefrontal cortex^59,60^, so it may integrate conceptual information into episodic memories^46^, and thus might be more related to gist-based details. ERC is also involved in encoding both temporal duration information^61^ (which is perception-based) and temporal sequence information^62–64^ (which is gist-based). Here we attempted to replicate previous associations between ERC thickness and temporal discounting, as well as to explore the relationship between perception-based and gist-based details and MTL structural integrity.

In addition to correlational evidence linking temporal discounting with episodic memory ability, previous research also shows that the most effective ways to *change* temporal discounting involve engaging the episodic memory system. Imagining positive future events decreases temporal discounting in young adults^16,17,65,66^, whether or not the future events are directly linked to the future rewards at stake^16^. Recently, we showed that positive autobiographical memory retrieval also reduces discounting in young adults^11^. Just as it is unknown which aspects of episodic memory are associated with temporal discounting across individuals, the mechanism by which memory-based manipulations increase patience also remains unclear. Previous research suggests that more vivid episodic imagery leads to more patient choice^16^, suggesting that memories richer in perception-based details might be more effective at changing choice, but previous studies have examined only self-reported ratings of vividness. Here we took advantage of our more objective autobiographical memory scoring analysis to investigate (1) whether recalling these autobiographical memories would influence intertemporal choice in older adults, and (2) which autobiographical details, if any, predict a change in temporal discounting after memory recall. We hypothesized that, as a group, older adults would show a blunted effect of memory recall on choice, due to their memory decline. Scoring descriptions of these memories, however, allowed us to investigate the extent to which any effect of the manipulation can be explained by inter-individual or intra-individual (i.e., between memories) variability in autobiographical details.

In sum, this study examines the following in a group of cognitively normal older adults: (1) the relationship between temporal discounting and autobiographical memory richness, (2) the relationship between both temporal discounting and autobiographical memory richness and medial temporal lobe structural integrity, and (3) whether retrieving positive autobiographical memories prior to choice impacts temporal discounting.

## Results

### Individual differences in perception-based autobiographical memory details are associated with temporal discounting

**Figure 1:**
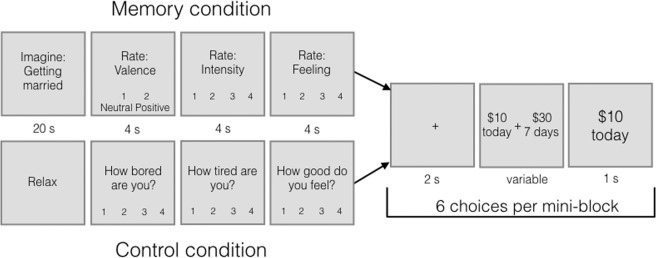
Task Layout. Each mini-block contained six intertemporal choices. Each memory mini-block began with a memory cue, describing an autobiographical memory specific to the participant. The participant was asked to think about that positive memory for 20 seconds. Then they rated the valence (1 = neutral; 2 = positive), intensity (1–4; 1 = not intense; 4 = very intense) and feeling (1–4; 1 = neutral; 4 = very good) of the memory. Finally, they made 6 choices between $10 today and a larger amount of money available after a delay. The participant made a button press while the options were on the screen, and then was shown what they chose for 1 s before the next trial began. In the Control mini-blocks, participants were told to relax for 20 s and then to answer questions about how bored and tired they were, and how good they felt (1–4 scale for each). They then made the same intertemporal choices in this condition. This procedure is adapted from^11^.

Thirty-four older adult participants (ages 65–90) completed a two-day task and were included in final analyses (see Methods for full demographic and exclusion information). On Day 1, they described twelve positive memories for four minutes each. These descriptions were audio recorded and scored using the Autobiographical Interview protocol^32^, yielding totals for internal event, time, place, perceptual, and emotion/thought details (Table 1). On Day 2, participants completed an intertemporal choice task in which, on each trial, they selected between $10 today and a larger amount of money available after a delay. In the Memory condition of that task, they recalled positive memories (the ones described on Day 1) before each set of six choices. In the Control condition, they simply relaxed prior to each set of six choices (see Fig. 1 for task layout).

**Table 1:** Descriptions, examples, and descriptive statistics of the categories of details that we related to temporal discounting rate in the current study. Scoring instructions courtesy of Brian Levine, Eva Svoboda, and Morris Moscovitch^32^. All details analyzed were internal to the event being described. That is, event, place, time, perceptual, and emotion/thought details that were not directly related to the event being described were not included in the tallies that were used in the perception-based detail ratio score analysis, although they were labeled and counted. The perception-based detail ratio score was computed (as in^35^) by taking the sum of time, place, and perceptual details, and dividing it by the total number of internal details.

Detail Category	Internal Detail Type	Description	Examples	Across-subject mean (SD), range
Perception-based	Place	Localization in space, including countries, bodies of water, cities, streets, buildings, rooms, locations within a room.	“It was down a little hill.” “Philadelphia” “St. Mary’s school”	2.42 (0.62) [1.33–3.33]
	Time	Life epoch, year, season, month, date, day of week, time of day, or clock time. Not duration information (perceptual) or information about sequences of events (event).	“My twenties” “It was February.” “In 8th grade”	2.43 (0.61) [0.89–3.56]
	Perceptual	Auditory, olfactory, tactile/pain, taste, visual (object details, colors), spatial-temporal (allocentric-egocentric space, body position and duration) information.	“Painted white railings” “I was in my seat.” “I felt hot.”	2.81 (1.30) [0.89–5.89]
Gist-based	Event	The unfolding of the story. Happenings, who was there, emotions in others, the weather, one’s clothing, physical occurrences and actions of others.	“He jumped out of the chair.” “It was sunny.” “My sister Sue was with me.”	11.49 (2.94) [4.67–18.33]
	Emotion / thought	Relating to the mental state of the subject at the time of the event. Feeling states, thoughts, opinions, expectations, beliefs.	“I was annoyed.” “I was quite excited.” “I assumed it was the other girl.”	4.40 (1.43) [1.56–8.67]

**Figure 2:**
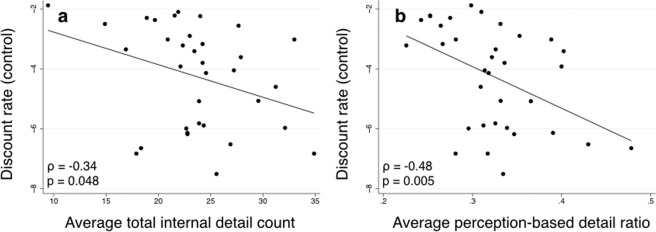
Association between average (**a**) count of total internal details, and (**b**) perception-based detail ratio across nine autobiographical memories for each participant and their temporal discounting rate. Temporal discounting rate in the Control condition of the intertemporal choice task was used as the dependent variable. Individuals who had more internal details in their memories overall, and those who mentioned relatively more time, place, and perceptual internal details (perception-based details) compared to event and emotion/thought internal details (gist-based details) were more likely to select larger, later rewards in the temporal discounting task.

Temporal discounting was correlated with the richness of autobiographical memories and the degree to which these memories contained perception-based, rather than gist-based, details. First, we found that lower discount rates (derived from the Control condition of the task) were associated with a greater number of internal, but not external, details in participants’ autobiographical memories (internal: ρ = −0.34; *p* = 0.048, Fig. 2a; external: ρ = −0.002; *p* = 0.990). Second, we found that lower discount rates were associated with a larger ratio of perception-based (time, place and perceptual) details compared to gist-based (event and emotion/thought) details (ρ = −0.48; *p* = 0.005; Fig. 2b). That is, individuals who recalled memories in a perceptually rich and specific spatiotemporal context were more likely to select larger, later rewards. This association between discount rate and perception-based detail ratio score was also significant when we used the Memory condition discount rate (ρ = −0.45; *p* = 0.007). This association was also robust to controlling for age, gender, years of education, and the average age of the participant’s memories (partial Pearson *r* = −0.44; *p* = 0.014). Remote memories have been shown to be more gist-based^36,46^, but here we did not find a relationship between the perception-based detail ratio and the age of the memory (*n* = 306 memories; *r* = −0.01; *p* = 0.824), perhaps because memories were, for the most part, remote (mean age of memory = 32.32 years; SD = 21.32 years). In a series of post-hoc correlations, we found that discounting was significantly correlated with each of the individual subcategories of perception-based details (time, place, and perceptual; see Supplementary Data).

To ensure that the relationship between perception-based autobiographical memory details and temporal discounting was not driven by differences in narrative style, we examined the association between subcategories of *external* details and temporal discounting. There was no evidence of any association between temporal discounting and external time details (ρ = −0.12; *p* = 0.514), external place details (ρ = 0.20; *p* = 0.269), external perceptual details (ρ = −0.16; *p* = 0.366), external event details (ρ = −0.04; *p* = 0.816), or external emotion/thought details (ρ = 0.22; *p* = 0.213). Discounting was also unrelated to subjective ratings of memories, and, in general, subjective ratings of memories were unrelated to objective measures of memory vividness (see Supplementary Data).

Consistent with previous research, the total number of internal details was associated with age, such that older individuals included fewer internal details overall in their memory descriptions (ρ = −0.35; *p* = 0.043). However, neither temporal discounting (ρ = 0.19; *p* = 0.293) nor the perception-based detail ratio score (ρ = −0.14; *p* = 0.434) was associated with age.

### Exploratory: entorhinal cortical thickness is associated with temporal discounting and perception-based autobiographical details

We previously found that temporal discounting was associated with structural integrity in ERC in older adults^31^. We had structural MRI data for a subset (*n* = 22) of the participants in the current study, which partially overlapped with our previous study. Considering that this is a small sample for examining individual differences in anatomical structure, but also the unique resource of the detailed scoring of autobiographical memory richness that we had for this sample, we conducted an exploratory analysis of the relationships between the structural integrity in MTL regions-of-interest (ROIs) and autobiographical memory details. Using an automatic segmentation pipeline, ASHS–T1^67^ (see Methods for details), we obtained volume measures for the anterior and posterior portions of the hippocampus, and mean cortical thickness in ERC, perirhinal cortex subregions Brodmann Area 35 (BA35) and Brodmann Area 36 (BA36), and parahippocampal cortex (PHC). We conducted a series of exploratory regression analyses to investigate relationships between thickness/volume in each of these subregions and temporal discounting, as well as our two measures of autobiographical memory richness: the average number of total internal details and the perception-based detail ratio score. As a control analysis, we also conducted these regressions with the average number of total external details.

**Figure 3:**
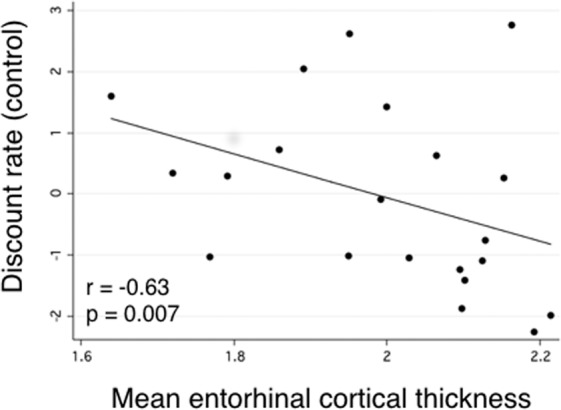
Individuals with more cortical thickness in entorhinal cortex displayed reduced temporal discounting, or a relatively greater preference for larger, delayed rewards (*n* = 21).

Consistent with our previous study^31^, temporal discounting was associated with mean ERC thickness (*r* = −0.63; *p* = 0.007; Fig. 3), but not thickness in BA35 (*r* = 0.09; *p* = 0.727), BA36 (*r* = 0.14; *p* = 0.585), or PHC (*r* = −0.33; *p* = 0.177), or anterior (*r* = −0.27; *p* = 0.287) or posterior hippocampal volume (*r* = −0.30; *p* = 0.245). We obtained the same results when using the discount rate in the Memory condition (ERC: *r* = −0.67; *p* = 0.003; BA35: *r* = 0.06; *p* = 0.816; BA36: *r* = 0.08; *p* = 0.752; PHC: *r* = −0.39; *p* = 0.113; anterior hippocampus: *r* = −0.31; *p* = 0.224; posterior hippocampus: *r* = −0.37; *p* = 0.142).

The average number of internal autobiographical details was associated with cortical thickness in ERC (*r* = 0.64; *p* = 0.006) and PHC (*r* = 0.63; *p* = 0.005), but only ERC thickness was additionally associated with the perception-based detail ratio score (*r* = 0.50; *p* = 0.042). This association was not driven by any particular subcategory of perception-based details, since time, place, and perceptual details were each associated with ERC thickness (see Supplementary Data). Notably, there was also a trend toward an association between the perception-based detail ratio score and the volume of the posterior hippocampus (*r* = 0.43; *p* = 0.086), the subregion of the hippocampus that has been proposed to be associated with context-rich episodic memories. There were no associations between external details and structural integrity of any MTL subregions. Table 2 presents the full list of partial correlation coefficients between autobiographical details and medial temporal lobe subregions.

**Table 2:** Associations between cortical thickness/volume in medial temporal lobe subregions and the average number of internal autobiographical details, external autobiographical details, and the perception-based detail ratio score. Note: Partial Pearson correlation coefficients, controlling for age, gender, years of education, and absolute number of days since MRI are shown. Analyses for hippocampal volume measures include total intracranial volume as an additional covariate. *p < 0.05, **p < 0.01 (uncorrected for multiple comparisons).

Medial temporal lobe region	Internal details	External details	Perception-based detail ratio
Entorhinal cortex	0.64**	0.21	0.50*
BA35	0.35	0.18	−0.22
BA36	0.003	0.18	0.22
Parahippocampal cortex	0.63**	−0.22	0.18
Anterior hippocampus (volume)	0.37	0.01	0.06
Posterior hippocampus (volume)	0.15	0.24	0.43

### No effect of positive memory retrieval on temporal discounting in older adults

**Figure 4:**
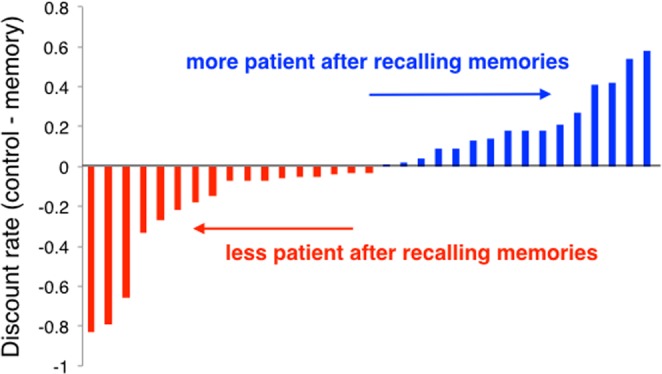
Positive memory retrieval does not affect intertemporal choice in older adults. The difference between the log-transformed discount rate in the Control condition and Memory condition is plotted for each subject. Positive difference (blue) indicates more patience in the positive memory condition. Negative difference (red) indicates more impulsivity in the positive memory condition. (*t*_33_ = −0.21; *p* = 0.834; *n* = 34; *n* = 1 not shown in figure because difference in discount rate was 0).

On Day 2 of the study, participants made a series of intertemporal choices either after retrieving positive autobiographical memories for 20 seconds (Memory blocks) or after relaxing for the same amount of time (Control blocks). Unlike in younger adults in a previous study^11^, the positive memory manipulation had no effect on temporal discounting in this older adult population (two-tailed *t*_33_ = −0.21; *p* = 0.834; Fig. 4). Surprisingly, on average, positive feeling ratings were not higher in the positive memory blocks compared to the control blocks (*t*_33_ = 1.09; *p* = 0.285), most likely because feeling ratings were close to ceiling in both conditions (mean feeling rating in Control blocks = 3.53; mean feeling rating in Memory blocks = 3.61; maximum feeling rating = 4).

Although recalling positive autobiographical memories did not have an overall effect on temporal discounting, recalling individual memories with a high percentage of internal details did bias choices towards delayed rewards. To investigate whether aspects of the memories themselves could predict the extent to which retrieving them was effective in reducing temporal discounting rate, we conducted two mixed-effects logistic regressions to see if (1) the percentage of internal details in the memory, or (2) the degree to which the memory contained more perception-based details, could predict choice of delayed reward on a trial-by-trial basis. Importantly, by entering the Control condition subjective value as a regressor in the model, this analysis controlled for the amount and delay offered on that trial, as well as the subject’s idiosyncratic discount rate in the Control condition (see Methods for details). We found that recall of memories that had relatively more internal details compared to external details was marginally more likely to lead to delayed reward choice (Coefficient = 1.48; *p* = 0.048). However, there was no effect of the perception-based detail ratio score on choice of the delayed reward on a trial-by-trial basis (Coefficient = −0.18; *p* = 0.848). Finally, there was no effect of the memory ratings made by participants (e.g., valence, vividness, intensity, positive feeling) on the extent to which recall of those memories impacted choice (see Supplementary Data).

We did not find any variables that could account for differences in the effect of memory retrieval on temporal discounting across individuals. While autobiographical memory details predicted temporal discounting rate at baseline, they were unrelated to the effect of memory retrieval on temporal discounting across individuals. That is, the difference in discount rate between Control and Memory conditions was not associated with the average number of internal details in participant memories (ρ = −0.12; *p* = 0.512) or with the average perception-based detail ratio score (ρ = −0.15; *p* = 0.413). The effect of memory retrieval on temporal discounting was also unrelated to structural integrity in any of the MTL regions that we examined (ERC: *r* = −0.05, *p* = 0.855; BA35: *r* = 0.22, *p* = 0.405; BA36: *r* = 0.41, *p* = 0.105; PHC: *r* = 0.19, *p* = 0.450; anterior hippocampus: *r* = 0.12, *p* = 0.647; posterior hippocampus: *r* = 0.29, *p* = 0.254). Finally, the effect of memory recall was not predicted by age (ρ = −0.15, *p* = 0.390).

## Discussion

In the current study, we examined associations between autobiographical memory richness and temporal discounting in a group of cognitively normal older adults. We found that the extent to which participants’ positive autobiographical memories contained perception-based, rather than gist-based, details was associated with individual differences in temporal discounting. People who were better able to localize their memories to a particular time and place, and who reported more sensations from that time and place, were more likely to choose larger, later rewards. In an initial exploratory analysis of a subset of participants with neuroanatomical data, we also found that cortical thickness in entorhinal cortex was associated with perception-based details as well as temporal discounting, suggesting that it may be a neural substrate connecting memory retrieval with decision making, a hypothesis that should be tested further in future research. We also tested whether recalling these positive autobiographical memories would reduce temporal discounting in older adults. Although recalling positive memories reduces temporal discounting in young adults^11^, it was not effective in older adults. However, within participants, the extent to which autobiographical memories contained a greater proportion of internal details overall predicted whether choices became more future-oriented following recall of those memories.

To our knowledge, this is the first study to detect an association between autobiographical memory details and temporal discounting. Previous studies found no association between overall autobiographical memory richness and discount rate^33,34^. By using a composite measure of internal (episodic) details, though, these studies may have masked the finding that discounting may be related to only select types of details. We did observe an association between the total number of internal details and temporal discounting here, but it was relatively weak and would not survive correction for multiple comparisons. A more important predictor of future-oriented decision making was the degree to which memories contained more perception-based details (i.e., time, place, and perceptual details). Importantly, *external* time, place, and perceptual details (those that are not directly related to the episode being described) were not associated with temporal discounting, showing that our results cannot be attributed to individual differences in narrative style. Perception-based details likely reflect episodic re-experiencing of an event and the mental construction of a specific scene. This finding advances our previous work, where we found an association between lower temporal discounting and better episodic memory abilities, but were unable to distinguish between different aspects of episodic recall^31^. Here we used a well-validated autobiographical memory scoring protocol that allowed us to categorize details into two qualitatively different categories and found that reduced discounting was linked to the details in autobiographical memory that are most purely episodic. An important caveat here, however, is that, even though it has been used this way before^35^, the Autobiographical Interview protocol was not initially designed to delineate between perception-based and gist-based details. Thus, it is possible that some details labeled as gist-based were actually perception-based, and *vice versa*. Future research using other autobiographical memory coding schemes will shed some additional light on this issue.

What could explain this association between more perceptually rich autobiographical memories and temporal discounting? Older adults with more perceptually rich mental recollections may also imagine the future in a more perceptually specific manner, which then leads them to perceive future rewards as more concrete or closer in time. This proposed mechanism is consistent with previous research showing that increasing the concreteness of delayed rewards (such as by pairing them with a specific spatial context, e.g., “spending $40 at a café”^16^) leads to more patient decision making.

In an exploratory analysis of the subset of participants who had structural neuroimaging data, we replicated the finding from our previous study^31^ that temporal discounting rate was associated with thickness in ERC (and no other subregion of the MTL). Though this sample partially overlaps with our prior study, it is notable that it included only cognitively normal participants, and that these participants performed a different intertemporal choice task from the previous study. When examining associations between autobiographical details and MTL structural integrity, we found that ERC thickness, like temporal discounting, was associated with both the number of internal details in memories and the extent to which memories were rich in perception-based details. This association is consistent with the well-known role of ERC in spatial navigation and memory^57,58^, and suggests the ERC may support temporal discounting by providing a spatiotemporal context for imagined future events. However, we hasten to add that these results should be interpreted with caution given the small sample (*n* = 21) and that they should be replicated in a larger study. Moreover, there is evidence that the medial and lateral portions of ERC play divergent roles in memory^62,68,69^, so it will be important for future studies to segment the ERC into these subsections.

Here we also tested if recalling positive memories directly prior to intertemporal choice would alter temporal discounting in older adults. We found no effect of recalling positive memories on temporal discounting in older adults, in line with a previous study finding no effect of episodic *future* thinking on temporal discounting in older adults^70^. Together, these studies suggest that memory-based manipulations have limited effectiveness in older adults, given that they show a marked decline in episodic memory^32,71,72^. Indeed, we found that retrieval of memories that contained relatively more internal details was more likely to increase patient choices. Compared to younger adults, older adults include fewer internal relative to external details in their autobiographical memory descriptions^32,38^, and this reduced level of detail may be the reason that the manipulation was ineffective for older adults. Patient choice was not influenced by any other aspects of the memories, including the relative richness in perception-based details and subjective reports of emotional intensity and vividness.

It is somewhat puzzling that whereas perception-based details were associated with temporal discounting rates at baseline, they did not predict whether memory recall influenced choice. There are a few possible explanations for this null finding. One is that since we controlled for baseline discounting in our analysis at the individual trial level, and perception-based details were strongly associated with baseline discounting, there was insufficient variance to detect any *additional* effect of perception-based details above and beyond that baseline effect. Another possibility is that processes contributing to stable time preferences may be distinct from those supporting the flexibility of choice at the time of the decision^73^. For example, individuals with hippocampal damage show similar temporal discounting rates compared to healthy controls^54^, but unlike healthy controls, their intertemporal choices are not affected by episodic future thinking^66^. A final possibility is that memory recall did not have a potent enough effect on intertemporal choice to reveal the effects of perception-based vs. gist-based details. Future studies could use episodic future thinking, a more reliable way to influence intertemporal choice, to test if imagining *future* events with more perception-based details is more likely to lead to patient intertemporal choices than imagining events with more gist-based details.

Some limitations of this study are worthy of mention. First, the sample size was chosen in order to detect the within-subject effect of autobiographical memory recall on temporal discounting, and therefore is small for examining individual differences (*n* = 34 overall; *n* = 22 in the neuroanatomical analyses). Thus, it is important not to over-interpret null results regarding individual differences, as there is a risk of Type-II error. This is particularly true for the neuroanatomical results. For example, while posterior hippocampus has been proposed to be associated with perception-based details^74,75^, here that association did not reach significance, which may be due to insufficient power. The neuroanatomical analyses in this paper are exploratory and meant to serve as a starting point for larger studies. Second, while we are confident that lower discounters have a greater proportion of perception-based details compared to gist-based details, inter-rater reliability was low for some detail types, and emotion/thought details may have been limited in number and variance since participants described only positive memories. Therefore, this finding should be replicated in follow-up studies with larger samples, a larger age range, and a more standard Autobiographical Interview paradigm. Finally, we did not include a young adult control group in this study, since our previous study^11^ used an almost identical paradigm to investigate the effects of positive memory recall on intertemporal choice. Therefore, the lack of effect of positive memory recall on intertemporal choice may not necessarily be due to aging; it is also possible that the effect of this manipulation is simply smaller than what was initially reported. We also cannot conclude that the associations found here will necessarily generalize to younger adult populations, but this is an important line of inquiry for further study.

In summary, here we showed, in a group of cognitively normal older adults, that autobiographical memory richness is correlated with temporal discounting. Perception-based details are associated with temporal discounting rates across individuals, and recalling memories that are richer in internal details prior to choice leads to more patient decisions. These findings will help to inspire and optimize interventions to nudge intertemporal choice, especially in older adults with more limited episodic memory ability. They also add to the growing literature on the critical role of episodic memory in making decisions about the future.

## Methods

### Participants

Thirty-eight older adult participants (ages 65–90; mean age = 74; SD = 6.9; see Supplementary Fig. S1 for histogram illustrating age distribution; 24 F, 14 M; 31 White, 6 Black, 1 Asian) completed this experiment. We selected thirty-eight as our target sample size as this would give us 80% power to detect an effect of Cohen’s *d* equal to 0.48, the effect size of positive autobiographical memory retrieval on temporal discounting in the young adult study we completed previously^11^. All subjects were deemed cognitively normal based on consensus diagnosis at the Penn Alzheimer’s Disease Core Center. Of the thirty-eight participants who completed the study, four were excluded. Three were excluded because their discount rates could not be estimated in one or both experimental conditions. Of these three, two chose all delayed rewards, and one chose all immediate rewards. An additional participant was excluded for having a score in the moderately depressed range on the Geriatric Depression Scale. Thus, thirty-four participants were included in final analyses (23 F; mean age = 74.11; SD = 6.97). All participants provided informed consent and were compensated $10/hour for their participation. The study was approved by the Institutional Review Board of the University of Pennsylvania (IRB #808893), and all research was conducted in accordance with the Declaration of Helsinki.

## Procedure

Participants completed a two-day study. On Day 1, they described positive memories prompted by each of 12 life event cues (e.g., “being in a wedding,” “winning an award”). The cues were a compilation of cues from prior studies^76,77^ and were designed to probe for positive memories.

Participants described only positive memories, since previous research has shown that positive, but not negative, memory recall reduces temporal discounting in young adults^11^. Thus, we decided to include only positive memories in order for this study to more closely match the previous one, and in order to maximize our chance to see an effect of our memory manipulation on temporal discounting in the second session.

For each cue, participants selected a memory in which they had been personally involved and that had occurred at a specific place and time. For each memory, participants had four minutes to provide a brief verbal description, and their response was audio recorded for later scoring with the Autobiographical Interview Protocol^32^ (see *Autobiographical memory scoring* section below). They were prompted when there was one minute left. Each memory cue was allotted four minutes, even if participants did not speak through the whole four minutes. An interviewer was present throughout this task, in order to prompt the participant for more detail if necessary. At the end of each memory description, they provided the location and date of the memory. Then, they gave subjective ratings for valence (1 = neutral; 2 = positive), emotional intensity (1–4: 1 = not intense, 4 = very intense), feeling now (i.e., how they felt when recalling the memory; 1–4: 1 = neutral, 4 = very good), feeling at the time of the memory (1–4: 1 = neutral, 4 = very good), personal importance of the memory (1–4: 1 = not important; 4 = extremely important), similarity between current self and self in the memory (1–4: 1 = very different; 4 = exactly the same) and vividness (1–4: 1 = not vivid, 4 = very vivid). A table with average memory ratings is presented in Supplementary Table S1. If participants could not think of a specific memory for a cue, or if the cue was only associated with negative memories, they could skip the cue and receive another. There were 24 possible cues, but the experiment terminated after twelve memories had been described (see Supplementary Methods in online supplemental materials for list of cues). This procedure was adapted from a previous study of episodic recall^78^.

In preparation for the second session, nine of each participant’s positive memories were selected. These nine had been rated as positive (i.e., valence = 2), and had the highest combined intensity and feeling ratings. They were summarized in subject-specific event cues that the participants reviewed at the beginning of the second session, to ensure that they could bring to mind the memory associated with each cue.

Participants returned for the second session about one week later (M = 7.15 days; SD = 1.85; range: 2–14 days) to perform an intertemporal choice task. On each trial of this task, they were presented with a screen showing two options: “$10 today” and a monetary reward of larger magnitude available after a delay (e.g., “$20 in 30 days”). Delayed reward amounts varied from $11 to $35, and delays varied from 1 day to 180 days (see Supplementary Methods in online supplemental materials for list of all amounts and delays). An effort was made to capture a range of hyperbolic discount rates (range: 0.00018–0.25) with the constraints that the immediate amount always be $10 and the delay not exceed 180 days. The immediate reward was kept constant, so that this paradigm closely resembled the previous version of this paradigm used in younger adults^11^. For each choice, participants made a button press, indicating which option they preferred (the task was self-paced). The order of the trials was randomized, and the immediate and delayed reward options switched sides of the screen randomly. After participants responded, they were shown the option they had just chosen for 1 second. After a 2 second inter-trial interval, the next choice screen appeared. There were 54 distinct choices, shown once in each condition, for a total of 108 trials.

Participants made these choices in two conditions, “Memory” and “Control,” presented in blocks. In Memory blocks, participants re-accessed the nine positive memories (yielding nine “mini-blocks”) triggered by cues from Day 1 before making choices. At the beginning of each Memory mini-block, a fixation point appeared for 3 seconds. Then, a memory cue was displayed for 20 seconds. Participants were asked to recall the memory described by this cue and to mentally elaborate on it for as long as they could or until 20 s were up. After a 3 s inter-stimulus interval, participants rated the memory on valence, emotional intensity, and feeling (allotted 4 s for each). Following this, participants made 6 intertemporal choices before the next memory cue appeared on the screen. The first memory block consisted of 5 mini-blocks (5 memories and 30 intertemporal choices), and the second memory block consisted of 4 mini-blocks (4 memories and 24 intertemporal choices; Fig. 1).

In each of the Control mini-blocks, participants first saw the word “Relax” on the screen for 20 s. They were instructed to rest during this time. Then, they rated how tired they were (1–4; 1 = very awake; 4 = very tired), how bored they were (1–4; 1 = not bored; 4 = very bored), and how good they felt (1–4; 1 = neither good nor bad; 4 = very good; 4 s for each rating). Following this, they made 6 intertemporal choices before the next “Relax” screen appeared. The first Control block consisted of 5 mini-blocks (5 “Relax” screens and 30 intertemporal choices), and the second Control block consisted of 4 mini-blocks (4 “Relax” screens and 24 intertemporal choices). There were two Control blocks and two Memory blocks, which alternated; the block type that came first was counterbalanced across subjects. We confirmed that there was no effect of which block type came first on the effect size of our manipulation (F_(1,32)_ = 0.65; *p* = 0.428). The same choices were presented in both conditions. The task was programmed using E-Prime 2.0 Stimulus Presentation Software (Psychology Software Tools, Sharpsburg, PA).

Participants were told at the outset of the Day 2 session that one of the intertemporal choice trials would be randomly selected and they would receive the amount they chose on that trial, at the delay specified. They were paid via a pre-paid debit card (Greenphire Clincard system). If they chose the immediate reward on that trial, they would receive the money on their card that day. If they chose the delayed reward, they would receive the money after the delay had elapsed. Paying out both reward types this way ensured that transaction costs were equivalent between immediate and delayed rewards.

After the decision-making task was completed, participants filled out four questionnaires on a computer: the Interpersonal Reactivity Index (IRI^79^), the Life Orientation Test-Revised (LOT-R^80^), the Geriatric Depression Scale (GDS^81^) and the Vividness of Visual Imagery questionnaire (VVIQ^82^). The GDS was included as a screening tool, because symptoms of depression are associated with deficits in memory ability, especially in positive memory recall^83^. Therefore, anyone with a GDS score of 9 or above (out of 15), indicating moderate or severe depression, was excluded (*n* = 1; see *Participants* above). Details of and results from analyses of these questionnaires can be found in Supplementary Methods, Supplementary Data, and Supplementary Fig. S2.

## Analyses

### Choice data

Participants’ individual intertemporal choice data were fit separately for choices in the Memory blocks and Control blocks with the following logistic function using maximum likelihood estimation:

$${P}_{1}=\frac{1}{1+{e}^{-\beta (SV1-SV2)}},\,{P}_{2}=1-{P}_{1}$$

Here, P_1_ refers to the probability of choosing the delayed option, and SV_1_ and SV_2_ are the subjective values of the delayed and immediate options, respectively. The subjective value of the options was assumed to follow a hyperbolic discounting function^9,84^:

$$SV=\frac{A}{1+kD}$$

where SV is the subjective value, A is the amount, D is the delay to receiving the reward, and *k* is the subject-specific discount rate parameter (higher *k* values correspond to more impatience). The hyperbolic function has been shown to fit temporal discounting data well^9,10^, including in older adults^85,86^. The model fit in the current study was good, with on average 93.57% (SD across subjects = 4.01%) of choices from the Control condition being accurately predicted by the SVs from the hyperbolic model (mean pseudo-R^2^ = 0.79, SD = 0.14). In the Memory condition, the model predicted 93.90% of choices (SD = 3.91%; mean pseudo-R^2^ = 0.81, SD = 0.12). There were no significant differences in model fit between conditions, whether we looked at pseudo-R^2^ (*t*_33_ = 1.07; *p* = 0.29) or percent choices predicted (*t*_33_ = 0.35; *p* = 0.73) as the model fit metric. The correlation between the Memory discount rate and Control discount rate was also very high (ρ = 0.98; *p* < 0.001). Since discount rates are not normally distributed, these parameters were log-transformed before statistical analyses were performed.

### Autobiographical memory scoring

Audio recordings from each of the nine memories used in the intertemporal choice task for each participant in the final sample (*n* = 34) were transcribed and then scored using the Autobiographical Interview protocol^32^. The transcription that was scored for each memory began after the participant selected the cue, and ended after the participant stated the date and location of the memory.

For each memory transcription, the central event was identified. If more than one event was mentioned, the event described in more detail (the same one also described by the cue on Day 2) was considered the main event. Each event was divided into distinct details (unique pieces of information), and these details were classified as internal to the event (episodic details) or external (information related to events other than the main event). Semantic information and repetitions were considered external. Internal details and external details that were not semantic details or repetitions were further categorized as: event details (describing happenings in the story), time details (pertaining to when the event occurred), place details (pertaining to where the event occurred), perceptual details (describing sensations during the event), and emotion/thought details (pertaining to the mental state of the subject at the time of the event; see Table 1 for more detail, examples, and descriptive statistics).

To quantify the extent to which each memory was richer in perception-based details relative to gist-based details, we used a previously defined metric^35^: the perception-based detail ratio score. This ratio is equal to the sum of the internal time, place, and perceptual details divided by the total number of internal details. Note that this ratio is the inverse of a gist-based detail ratio score.

Two raters who were blind to the hypothesis of the study scored the transcripts independently. A main rater scored all memories, with the second rater scoring approximately half of them (135 memories from 15 participants). Inter-rater reliability for total internal and external details in each memory was assessed using the intra-class correlation coefficient (ICC^87^). The ICC was 0.48 for internal details (two-way ANOVA with raters as fixed effects: F_(134,134)_ = 2.8; *p* < 0.001, 95% CI: [0.36, 0.58]) and 0.57 for external details (F_(134,134)_ = 3.7; *p* < 0.001, 95% CI: [0.47, 0.66]). For each category of internal details, the ICCs were as follows: 0.51 for place details (F_(134,134)_ = 3.1; *p* < 0.001, 95% CI: [0.4, 0.61]), 0.51 for time details (F_(134,134)_ = 3.1; *p* < 0.001, 95% CI: [0.4, 0.61]), 0.34 for event details (F_(134,134)_ = 2.03; *p* < 0.001, 95% CI: [0.21, 0.46]), 0.23 for perceptual details (F_(134,134)_ = 1.57; *p* = 0.003, 95% CI: [0.10, 0.36]), and 0.36 for emotion/thought details (F_(134,134)_ = 2.1; *p* < 0.001, 95% CI: [0.23, 0.48]).

These ICCs were low compared to some previous studies^32,33^. We believe that this may stem from the main rater having more training in the Autobiographical Interview scoring protocol than the second rater (the main rater scored 20 practice memories, whereas the second rater scored 10 memories). The main rater adhered closely to the original scoring protocol, with the average correlation between their detail counts matching the detail counts for the practice memories provided by three scorers from the Levine lab (total details: *r* = 0.96; internal details: *r* = 0.84; external details: *r* = 0.98). These correlation coefficients were somewhat lower for our second rater (total details: *r* = 0.95; internal details: *r* = 0.61; external details: *r* = 0.89), suggesting that the second rater’s classification of internal vs. external details likely deviated slightly from the original scoring protocol.

Although ICCs were relatively lower for some detail types, we do not believe that our results are driven by some details being more reliably scored than others. First, ICCs were not systematically lower for the perception-based details (time, place and perceptual) compared to the event-based details (event and emotion/thought). Second, for the subset of participants (*n* = 15) that were scored by both raters, we did not find any evidence that our primary measure, the perception-based detail ratio score, was influenced by reliability. We computed the perception-based detail ratio for each memory separately for each scorer, and then found the Pearson correlation between scorers for this measure for each participant. There was no relationship between the reliability of a participant’s perception-based detail score and the score itself (*r* = −0.08; *p* = 0.782), nor was there a relationship between reliability and temporal discounting rate (*r* = −0.10; *p* = 0.72).

### Autobiographical details and individual differences in discount rate

Scoring autobiographical details allowed us to examine the relationship between autobiographical memory richness and temporal discounting across participants. For each participant, we averaged the following across memories: (1) the total number of internal details, (2) the total number of external details, and (3) the perception-based detail ratio score. Spearman correlations were conducted between the Control condition discount rate and each of these measures. We hypothesized that temporal discounting would be associated with the total number of internal details, but not external details. Thus, the external detail analysis served as a control for participant verbal output.

In addition, we explored the relationship between age and temporal discounting, as well as between age and internal details, external details, and the perception-based detail ratio. We expected to find no relationship between age and temporal discounting, consistent with previous work^31^.

Finally, we examined how individual differences in average participant ratings of their memories (from both Day 1 and Day 2) related to temporal discounting, as well as to the objective measures of memory richness described above. Results from these analyses are presented in Supplementary Data.

### Structural MRI data acquisition and analysis

Twenty-two participants in the sample also underwent MRI scanning within approximately one year of participating in this experiment (mean number of days between MRI and first behavioral testing session = 213.55; SD = 112; range: [14, 405]). MRI data were obtained on a Siemens Prisma 3T MRI with a 64-channel head coil. T1-weighted high-resolution magnetization-prepared rapid-acquisition gradient echo (MPRAGE; 0.8 × 0.8 × 0.8 mm^3^ voxels; TR/TE/TI = 1600/3.87/950 ms; flip angle = 15°) anatomical scans were collected. The medial temporal lobe was segmented using an automatic pipeline, ASHS–T1^67^. This technique uses a multi-atlas label fusion approach^88^ together with a tailored segmentation protocol to take into account anatomical variability in MTL cortex. It also explicitly labels dura, which has similar appearance to gray matter in T1-weighted MRI, resulting in more accurate segmentation of MTL cortex compared to other T1-MRI segmentation pipelines, such as FreeSurfer^67^. In addition to separate volume measures for the anterior and posterior hippocampus, we obtained measures of mean cortical thickness in the following regions-of-interest: entorhinal cortex, perirhinal cortex subregions BA35 and BA36, and parahippocampal cortex, using a graph-based multi-template thickness analysis pipeline^89^. We conducted a series of exploratory regression analyses to investigate relationships between thickness/volume in each of these subregions and temporal discounting, as well as with the average number of internal details, external details (as a control analysis), and the perception-based detail ratio score. Thickness and volume measures were averaged across hemispheres. Image quality was inadequate for segmentation of ERC, BA35, and BA36 for one participant (leaving *n* = 21 for those analyses). For another participant, image quality was inadequate for BA36 on one side only, so the mean was replaced with mean cortical thickness from the available side. Age, gender, years of education, and the absolute number of days between the MRI scan and the first behavioral testing session were entered as covariates of no interest. For the hippocampal volume analyses, we included the (square root-transformed) intracranial volume as an additional covariate. Partial Pearson correlation coefficients are reported.

### Positive autobiographical memory manipulation analysis

To test for the effect of our positive memory manipulation on temporal discounting overall, we conducted a two-tailed paired *t*-test to compare discount rates between Memory and Control conditions for each participant. We also investigated which, if any, details of the memories themselves predicted the extent to which they were effective in reducing discount rate. To this end, we conducted two mixed-effects logistic regressions predicting choice (0 = chose $10 immediate reward; 1 = chose delayed reward) on all trials in the Memory condition. First, we looked at the *percentage* of internal details relative to the total (internal + external) number of details for that memory (since memory descriptions vary with respect to total verbal output). Second, we used the memory’s perception-based detail ratio score as an independent variable. In both regressions, we controlled for the subjective value of the delayed option on that trial, assuming the participant’s discount rate from the Control condition. Specifically, we plugged in the discount rate *k* that was fitted to the data in the Control condition only, along with the amount and delay on that particular trial into the hyperbolic model equation, to determine the subjective value of the delayed reward on that trial, and entered this as a nuisance regressor. This is a conservative test that allowed us to see whether autobiographical details could predict delayed reward choice in the Memory condition *above and beyond* what could be predicted from the discount rate in the Control condition trials alone. We allowed slopes (for the regressor of interest) and intercepts to vary by subject. We conducted a similar analysis with the participants’ ratings of their memories as independent variables, yielding null results. The results of that analysis are presented in Supplementary Data in the online supplemental materials.

In addition to examining within-subject variance in the effect of memory recall on discounting, we also examined whether individual differences in autobiographical details (i.e., average total internal details, external details, and perception-based detail ratio), differences in age, or differences in MTL subregion thickness/volume predicted the effect of memory recall (difference between log-transformed discount rate in Control condition and Memory condition) across participants.

## Supplementary information

Supplementary Information.
