# Supplementary material for: Links between autobiographical memory richness and temporal discounting in older adults
Source: Sci Rep. 2020 Apr 14;10:6431. doi: 10.1038/s41598-020-63373-1 (PMC7156676; doi:10.1038/s41598-020-63373-1)
Supplement: Supplementary file 1 — Supplementary Information. [file 41598_2020_63373_MOESM1_ESM.pdf]

Supplementary Information for:

# Links between autobiographical memory richness and temporal discounting in older adults

Karolina M. Lempert[1], Kameron A. MacNear[1], David A. Wolk[2], Joseph W. Kable[1*]

[1]Department of Psychology, University of Pennsylvania.

[2]Hospital of the University of Pennsylvania, University of Pennsylvania.

[*]Correspondence concerning this article should be addressed to Joseph W. Kable, 433 S. University Avenue, Goddard 5, Philadelphia, PA 19104. Phone: 215-746-3873. E-mail: kable@psych.upenn.edu

## Supplementary Methods

*Self-report questionnaires*

After the decision making task was completed, participants filled out four questionnaires on a computer: the Interpersonal Reactivity Index (IRI), the Life Orientation Test-Revised (LOT-R), the Geriatric Depression Scale (GDS), and the Vividness of Visual Imagery questionnaire (VVIQ). The IRI assesses perspective-taking and empathy abilities, abilities that have recently been shown to be associated with temporal discounting[1]. The IRI has four subscales: Perspective-Taking, Empathic Concern, Personal Distress, and Fantasy. We focused analyses on the IRI Perspective-Taking subscale, as this was the one we expected to be most strongly related to temporal discounting based on previous research. The LOT-R tests for optimism, which tends to be elevated in older adults[2], and may be related to future-oriented decision-making[3]. The GDS was included as a screening tool, because symptoms of depression are associated with deficits in memory ability, especially in positive memory recall[4]. Therefore, anyone with a GDS score of 9 or above (out of 15), indicating moderate or severe depression, was excluded. Finally, the VVIQ instructs participants to imagine different scenarios in order to measure individual differences in self-reported imagery vividness. VVIQ scores have been shown to be correlated with temporal discounting[5]. We examined Spearman correlations between scores on these questionnaires and age, as well as with the size of the effect of the positive memory recall manipulation in our study. Outliers (scores that were more than 2.5 SD from the mean) were removed. There was 1 outlying VVIQ score (3.16 SD below the mean), and 1 outlying IRI-perspective-taking score (3.17 SD below the mean), leaving $n = 33$ for those analyses.

*List of cues used to elicit positive memory recall on Day 1*

Visit from friend/relative from out of town
Participating in sports
Graduating
Birth of a child
Going to the beach
Being in a wedding
Getting engaged or married
Going to a museum
Going to a concert
Going to the theater
Getting a pet
Hosting a party
Favorite team winning a championship
A friend's birthday party
Fourth of July
Thanksgiving
Winning an award
Getting a job/college/program acceptance letter
Being on a ship/boat
Memorable meal
Class or family reunion
Being promoted/given a raise
Camping or hiking
Buying a house or apartment

*List of choice sets in the intertemporal choice task (immediate reward amount was always $10).*

| Delayed amount ($) | Delay (days) |
|---|---|
| 17 | 1 |
| 20 | 2 |
| 18 | 3 |
| 20 | 4 |
| 24 | 5 |
| 27 | 6 |
| 13 | 7 |
| 14 | 7 |
| 16 | 8 |
| 19 | 9 |
| 25 | 10 |
| 29 | 11 |
| 14 | 13 |
| 25 | 15 |

| | |
|---|---|
| 19 | 17 |
| 26 | 18 |
| 16 | 20 |
| 12 | 24 |
| 15 | 26 |
| 32 | 27 |
| 21 | 31 |
| 28 | 38 |
| 15 | 42 |
| 13 | 59 |
| 34 | 60 |
| 35 | 62 |
| 22 | 67 |
| 11 | 74 |
| 18 | 75 |
| 33 | 76 |
| 13 | 86 |
| 12 | 90 |
| 18 | 91 |
| 12 | 96 |
| 15 | 100 |
| 11 | 105 |
| 32 | 107 |
| 11 | 117 |
| 15 | 126 |
| 24 | 127 |
| 14 | 129 |
| 12 | 135 |
| 11 | 137 |
| 12 | 138 |
| 19 | 141 |
| 14 | 154 |
| 25 | 158 |
| 11 | 166 |
| 11 | 167 |
| 19 | 171 |
| 11 | 173 |
| 13 | 176 |
| 12 | 179 |
| 11 | 180 |

**Supplementary Data**

*Temporal discounting and entorhinal cortical thickness are associated with each sub-category of perception-based details*

To see if any particular subcategories of internal details were driving the relationship between the perception-based detail ratio and temporal discounting, we conducted a series of post-hoc correlations. Discount rate was associated with all of the perception-based details – time details ($\rho = -0.46$; $p = 0.006$), place details ($\rho = -0.54$; $p < 0.001$), and perceptual details ($\rho = -0.36$; $p = 0.039$). In contrast, discount rate was not associated with any of the gist-based details – neither event details ($\rho = -0.08$; $p = 0.649$) nor emotion/thought details ($\rho = -0.13$; $p = 0.475$).

We found a similar pattern for entorhinal cortex (ERC) thickness. ERC thickness was significantly associated with the average number of time ($r = 0.58$; $p = 0.014$), place ($r = 0.71$; $p = 0.002$), and perceptual details ($r = 0.62$; $p = 0.008$), but not event ($r = 0.26$; $p = 0.322$) or emotion/thought details ($r = 0.37$; $p = 0.143$).

*Participant ratings of memories were not associated with temporal discounting*

Although objective assessments of autobiographical memories were associated with temporal discounting, none of the participants' subjective ratings of their memories were significant predictors of temporal discounting: there was no association with the Day 1 rating of "similarity between past and present self" ($\rho = 0.08$; $p = 0.661$), the Day 1 rating of "feeling when recalling the memory now" ($\rho = -0.15$; $p = 0.410$), the Day 1 rating of "emotional intensity" ($\rho = -0.03$; $p = 0.883$), the Day 1 rating of "feeling during the memory" ($\rho = -0.10$; $p = 0.572$), the Day 1 rating of "personal importance of the memory" ($\rho = 0.21$; $p = 0.239$), the Day 1 rating of memory vividness ($\rho = 0.05$; $p = 0.770$), the Day 2 rating of valence ($\rho = -0.10$; $p =$

0.570), the Day 2 rating of emotional intensity ($\rho = 0.07$; $p = 0.681$), or the Day 2 rating of "feeling when recalling the memory now" ($\rho = 0.15$; $p = 0.420$).

*Subjective measures of memory vividness are not associated with objective measures of autobiographical memory richness*

Participants rated their memories on several dimensions after describing them. To examine if any of these subjective ratings were associated with objective measures of memory richness, we conducted a series of regressions (clustering standard errors by subject) between each memory's rated vividness, emotional intensity, personal importance, feeling during recall, feeling during the memory, and felt similarity between past and present self and (1) the total number of internal details, and (2) the perception-based detail ratio score. There were no significant relationships between the number of internal details and the ratings ($n = 306$ memories from $n = 34$ subjects; vividness: $\beta = 0.48$; $p = 0.617$; intensity: $\beta = -0.47$; $p = 0.552$; feeling during recall: $\beta = -0.56$; $p = 0.460$; feeling during the memory: $\beta = -1.18$; $p = 0.071$; similarity: $\beta = -0.55$; $p = 0.438$), with the exception that memories of events that were rated as more personally important contained more internal details ($\beta =-1.51$; $p = 0.018$). Subjective ratings of memories were also not associated with the perception-based detail ratio (vividness: $\beta = -0.02$; $p = 0.108$; intensity: $\beta = -0.01$; $p = 0.206$; feeling during recall: $\beta = -0.002$; $p = 0.899$; feeling during the memory: $\beta = 0.01$; $p = 0.349$; similarity: $\beta = -0.007$; $p = 0.378$; personal importance: $\beta = -0.01$; $p = 0.231$).

*Participant ratings of memories did not predict change in choice following recall of memories*

We investigated whether any subjective characteristics of the memories themselves, as rated by the participants, could predict the extent to which retrieving them was effective in reducing temporal discounting rate. We conducted a series of mixed-effects logistic regressions to see which ratings could predict choice of delayed reward on a trial-by-trial basis, controlling for the subjective value of rewards computed assuming the Control condition discount rate. None of the ratings were significant predictors of choice: there was no effect of the Day 1 rating of "similarity between past and present self" (Coefficient = -0.028; $p = 0.792$), the Day 1 rating of "feeling when recalling the memory now" (Coefficient = -0.092; $p = 0.588$), the Day 1 rating of "emotional intensity" (Coefficient = -0.184; $p = 0.179$), the Day 1 rating of "feeling during the memory" (Coefficient = -0.330; $p = 0.056$), the Day 1 rating of "personal importance of the memory" (Coefficient = -0.087; $p = 0.470$), the Day 1 rating of memory vividness (Coefficient = 0.118; $p = 0.476$), the Day 2 rating of valence (Coefficient = 0.099; $p = 0.893$), the Day 2 rating of emotional intensity (Coefficient = 0.125; $p = 0.410$), or the Day 2 rating of "feeling when recalling the memory now" (Coefficient = -0.048; $p = 0.771$).

*Self-reported perspective-taking is associated with reduction in discounting following memory recall across participants*

The VVIQ, which measures individual differences in self-reported imagery vividness, was not correlated with temporal discounting rate ($n = 33$, 1 outlier excluded; $\rho = -0.09$; $p = 0.627$) or with the effect of memory recall on temporal discounting ($\rho = -0.03$; $p = 0.883$). However, VVIQ was associated with age ($\rho = -0.71$; $p < 0.001$) in the expected direction, with younger age associated with more vivid mental imagery. With respect to autobiographical

memory details, VVIQ was associated with the overall number of internal details ($\rho = 0.37$; $p = 0.033$), but not the perception-based detail ratio score ($\rho = -0.02$; $p = 0.903$).

The LOT-R, which measures optimism, was associated with the effect of memory recall at a trend level ($n = 34$; $\rho = 0.30$; $p = 0.088$). LOT-R scores were not associated with age ($\rho = -0.06$; $p = 0.721$), Control condition temporal discounting rate ($\rho = 0.03$; $p = 0.884$), number of total internal details ($\rho = 0.07$; $p = 0.681$), or the perception-based detail ratio score ($\rho = 0.03$; $p = 0.847$).

The IRI perspective-taking subscale measures an individual's tendency to consider the perspective of others in everyday situations. There was a significant association between an individual's perspective-taking score and the effect of memory recall ($n = 33$; 1 outlier excluded; $\rho = 0.60$; $p < 0.001$; Supplementary Fig. S2), in that individuals with a greater propensity and/or capacity to take the perspective of others were more likely to show reduced temporal discounting after recalling positive memories. This association was robust to controlling for the covariates of age, gender, and years of education (partial $\rho = 0.55$; $p = 0.002$). Perspective-taking was not related to temporal discounting rate in the Control condition ($\rho = 0.14$; $p = 0.453$), number of internal autobiographical memory details ($\rho = -0.006$, $p = 0.972$), or the perception-based detail score ($\rho = 0.08$, $p = 0.643$).

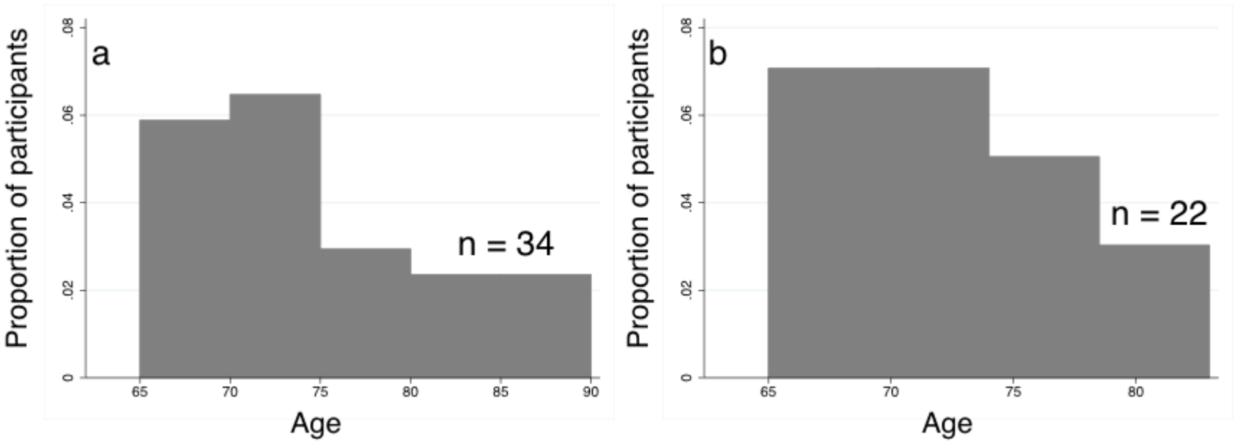


**Supplementary Fig. S1.** Histograms showing distribution of ages in full sample (a) and neuroimaging-only sample (b).

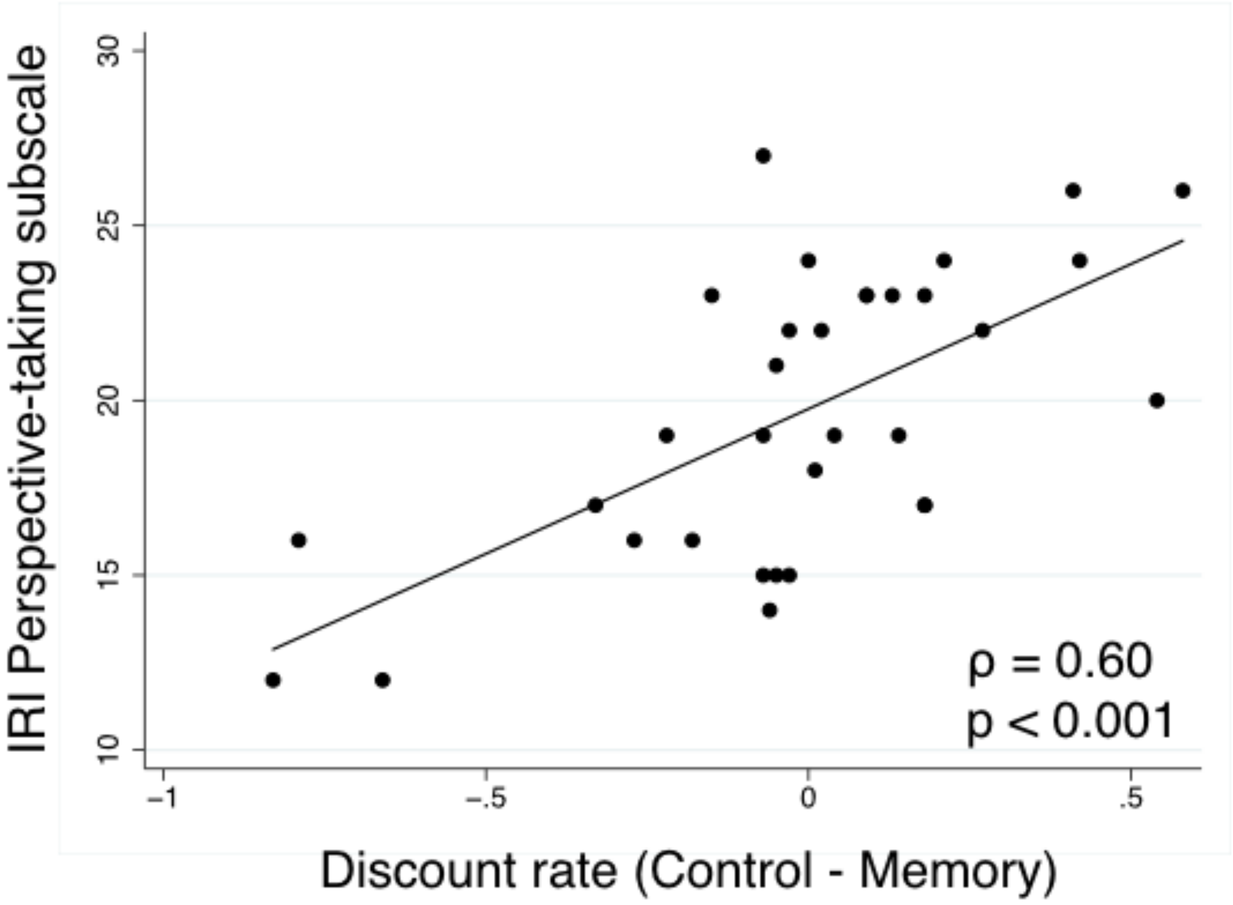


**Supplementary Fig. S2.** The perspective-taking subscale of the Interpersonal Reactivity Index was significantly associated with the degree to which discount rates were reduced after participants recalled positive memories.

| Rating (Average over all 9 memories) | Mean, SD, range |
|---|---|
| Day 2 Valence (1 = neutral; 2 = positive) | 1.98, 0.05, 1.78-2 |
| Day 2 Feeling now | 3.61, 0.41, 2.78-4 |
| Day 2 Emotional intensity | 3.18, 0.55, 2.11-4 |
| Day 1 Feeling now | 3.65, 0.30, 2.89-4 |
| Day 1 Emotional intensity | 3.34, 0.51, 2.11-4 |
| Day 1 Feeling at time of memory | 3.72, 0.31, 2.67-4 |
| Day 1 Personal importance | 3.30, 0.53, 2.33-4 |
| Day 1 Similarity between past and current self | 2.87, 0.64, 1.44-4 |
| Day 1 Vividness | 3.55, 0.34, 2.89-4 |

**Supplementary Table S1**. Average ratings of memories recalled during intertemporal choice task. Note: With the exception of valence, the scale for each rating was 1-4. Mean, SD, and range are across participants. Valence from Day 1 is excluded, since memories chosen for the task on Day 2 had a valence of 2.

**Supplementary References**


1. Soutschek, A., Ruff, C. C., Strombach, T., Kalenscher, T. & Tobler, P. N. Brain stimulation reveals crucial role of overcoming self-centeredness in self-control. *Sci. Adv.* **2**, e1600992 (2016).
2. Mather, M. & Carstensen, L. L. Aging and motivated cognition: the positivity effect in attention and memory. *Trends Cogn. Sci.* **9**, 496–502 (2005).
3. Berndsen, M. & van der Pligt, J. Time is on my side: optimism in intertemporal choice. *Acta Psychol. (Amst).* **108**, 173–86 (2001).
4. Dillon, D. G. The neuroscience of positive memory deficits in depression. *Front. Psychol.* **6**, 1295 (2015).
5. Parthasarathi, T., McConnell, M. H., Luery, J. & Kable, J. W. The Vivid Present: Visualization Abilities Are Associated with Steep Discounting of Future Rewards. *Front. Psychol.* **8**, 289 (2017).
